# Extracting conformational structure information of benzene molecules via laser-induced electron diffraction

**DOI:** 10.1063/1.4952602

**Published:** 2016-05-24

**Authors:** Yuta Ito, Chuncheng Wang, Anh-Thu Le, Misaki Okunishi, Dajun Ding, C. D. Lin, Kiyoshi Ueda

**Affiliations:** 1 Institute of Multidisciplinary Research for Advanced Materials, Tohoku University, Sendai 980-8577, Japan; 2 Institute of Atomic and Molecular Physics, Jilin University, Changchun 130012, People's Republic of China; 3 J. R. Macdonald Laboratory, Department of Physics, Kansas State University, Manhattan, Kansas 66506-2604, USA

## Abstract

We have measured the angular distributions of high energy photoelectrons of benzene molecules generated by intense infrared femtosecond laser pulses. These electrons arise from the elastic collisions between the benzene ions with the previously tunnel-ionized electrons that have been driven back by the laser field. Theory shows that laser-free elastic differential cross sections (DCSs) can be extracted from these photoelectrons, and the DCS can be used to retrieve the bond lengths of gas-phase molecules similar to the conventional electron diffraction method. From our experimental results, we have obtained the C-C and C-H bond lengths of benzene with a spatial resolution of about 10 pm. Our results demonstrate that laser induced electron diffraction (LIED) experiments can be carried out with the present-day ultrafast intense lasers already. Looking ahead, with aligned or oriented molecules, more complete spatial information of the molecule can be obtained from LIED, and applying LIED to probe photo-excited molecules, a “molecular movie” of the dynamic system may be created with sub-Ångström spatial and few-ten femtosecond temporal resolutions.

## I. INTRODUCTION

Imaging the real time evolution of a chemical reaction or a biological function is one of the major frontier research goals in modern science. For such purpose, it requires the development of new tools that are capable of probing molecules with a temporal resolution of a few to tens of femtoseconds and a spatial resolution of a few sub-Ångströms.^1–3^ These tools would enable the observation of changes of molecules near the conical intersection,^4^ the presence of transition states,^5^ and the ability to follow the dynamics of chemical processes such as proton migration,^6^ roaming,^7^ and ring opening,^8^ thus to create a “molecular movie”^2^ of the motions of atomic constituents. Understanding chemical reactions or biological functions at such a fundamental level would further open up future capabilities for creating conditions to drive the desired chemical processes that lead to favored irreversible chemical products.

For many decades conventional X-ray^9^ and electron^10^ diffraction methods have been routinely used to determine atomic positions in a molecule or in matter with sub-Ångström spatial resolution. However, these conventional sources are not suitable for probing dynamics of molecules that occur at femtoseconds timescale. An obvious approach for the new desired dynamic imaging tools is to extend these X-rays and electron pulses to timescale of a few to tens of femtoseconds. Indeed such intense femtosecond X-ray free-electron lasers (XFELs) are now available in the US^11^ and Japan,^12^ and a few more are coming out in other countries. These are costly large-scale national facilities, and they would not become tools for general purpose chemistry laboratories. At present, the XFELs still have to deal with spectrotemporal jitters and the energy is still too low to achieve sub-Ångström spatial resolution.^8,13^ Similar efforts have also been devoted to tools for ultrafast electron diffraction (UED). The achievable temporal resolution for electron pulses is currently still limited to about hundreds of femtoseconds for condensed matter applications,^2^ and about one picosecond for gas-phase molecule applications.^14^ Very recently, development of electron pulses with ∼30 fs duration^15^ and a novel dense pulsed electron source^16^ has been reported. Efforts are continuing aiming at reducing the space charge effects or using relativistic electron bunches.^17,18^ In other words, tools for ultrafast X-ray or ultrafast electron diffraction methods for dynamic imaging of molecules are still not yet fully developed, especially for the purpose of imaging chemical reactions in the gas phase.

In the past decade, alternative tools for dynamic imaging have also been conceived, especially for ultrafast electron diffraction. These include inner-shell photoelectron diffraction (ISPED),^19,20^ laser-assisted electron diffraction (LAED),^21^ and laser-induced electron diffraction (LIED).^22–24^ The femtosecond temporal resolution for ISPED is provided by the XFELs and for LIED is provided by femtosecond mid-infrared lasers, or its optical cycles (see below), while for LAED it is by the laser beam that intersects with the electron beam in conventional electron diffraction arrangement. Among all of these methods, UED, LAED, and LIED have demonstrated sub-Ångström spatial resolutions and they have the potential of becoming tools for dynamic imaging at individual laboratories. None has truly successfully applied to dynamic systems with temporal resolutions of a few to tens of femtoseconds yet except in LIED when combined with a theoretical modeling.^23^

**FIG. 1.**
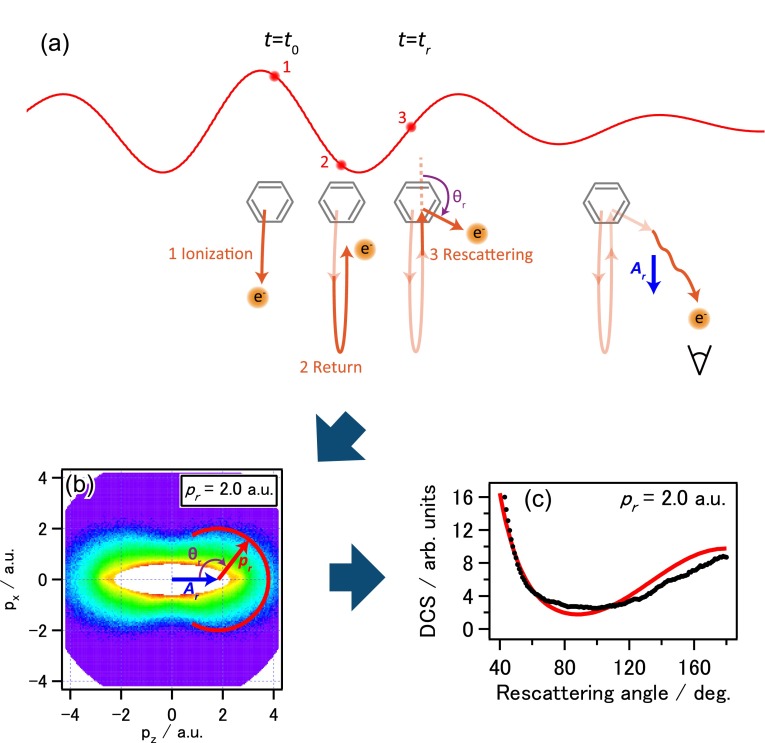
Extraction of field-free electron-ion elastic scattering DCS from 2D electron momentum distribution in laser induced electron diffraction. (a) Schematics of a tunnel-ionized electron driven back by the strong laser field to recollide with the parent ion, to generate electron diffraction image. (b) Typical experimental 2D electron momentum distribution of a benzene molecule. The electron-ion DCS at a fixed momentum *p_r_* is extracted along the red circle indicated. (c) Comparison of extracted molecular DCS from the 2D momentum distribution (black) and the calculated atomic DCS (red). Analysis of the difference between the two curves allows the retrieval of the bond lengths of atoms in the molecule.

The LIED is based on probing the structure of a molecule using its own electrons. When a molecule is placed in a femtosecond laser field, see Fig. 1(a), an electron may be tunnel ionized near the peak electric field of the laser's optical cycle (step 1). If these freed electrons go directly to the detector, they will appear as low-energy electrons, with a maximal energy of about 2*U_p_*, where *U_p_* is the quiver energy, or the ponderomotive energy, of a free electron in the laser field. *U_p_* is proportional to the laser intensity and to the square of the laser wavelength. However, after tunnel ionization, a fraction of these electrons may be driven back, at step 2 [see Fig. 1(a)], to return to collide with the parent ion, at step 3 [see Fig. 1(a)]. The maximal kinetic energy of these returning electrons can be calculated to be about 3*U_p_*. If these electrons are forwardly scattered, they will be slowed down by the succeeding field of the laser. These low-energy electrons are not of interest for LIED for structure retrieval. If these returning electrons are backscattered, they will be accelerated by the remaining field of the laser and emerge as high energy electrons. For such a large-angle scattering to occur, the returning electrons will have to come close to the atomic centers of the molecule. The diffraction image from the interference of collisions from these atomic centers is used to retrieve the interatomic bond lengths of the molecule. In Fig. 1(b), the red partial circle depicts the location of the diffraction image for a returning electron with momentum *p_r_*, elastically backscattered by large angles. The center of this partial circle is shifted by a vector $A_{r}$, which is directly related to the vector potential of the laser pulse at the time of collision (*t* = *t_r_*). This is the additional momentum gained by the scattered electron after it exits from the laser field. The description of the recollision above is given by the quantitative rescattering (QRS) theory.^25,26^ The QRS allows the extraction of field-free elastic electron-ion scattering differential cross section (DCS) with momentum *p_r_*, to be read off directly from the red circle, which is shown in the black dots in Fig. 1(c) after a normalization. The validity of the QRS approach has been previously checked for atomic targets experimentally.^27,28^

In conventional electron diffraction, molecular bond lengths are extracted from the DCS at small angles by colliding molecules with high energy electrons. High energy electrons have small de Broglie wavelength for better spatial resolution but, more importantly, the DCS described by the Born approximation^29^ can be readily inverted to obtain molecular bond length vectors. In electron diffraction, the Born approximation is replaced by the more accurate Independent Atom Model (IAM).^30^ This model can also be easily inverted to obtain bond length vectors. To apply IAM to LIED, there are a few limitations. First, a typical Ti-Sapphire laser will give maximal return electron energies of about 30 eV, which is far less than the tens or hundreds keV electrons used in the conventional electron diffraction (CED) experiment. Fortunately, as shown in Xu *et al.*,^22^ for IAM to work, one does not need keV electrons. The IAM can accurately describe the DCS in electron collisions with nitrogen or oxygen molecules using 100 eV electrons, if the DCS is taken at large scattering angles. At such energies, the spatial resolution was found^22^ to be about 5 pm. In Born approximation, the DCS depends on the momentum transfer **s** only,^29^ where the magnitude s=2k sin(θ/2). For CED, the momentum *k* is large but the scattering angle *θ* is small. For LIED, *k* is small but *θ* is large. Thus, the two methods cover the same range of momentum transfer, or in both cases, they cover about the same range of impact parameters in the collision. To obtain 100 eV returning electrons for LIED experiments, mid-infrared lasers with wavelength near 2 *μ*m can be used. Longer wavelengths will be needed for molecules with a low ionization potential to avoid excessive multiple ionization of molecules.

While the concept of LIED was conceived and demonstrated earlier,^31,32^ the first LIED experiment achieving sub-Ångström spatial resolution was reported by Blaga *et al.*^23^ Using 2 *μ*m mid-infrared laser, they were able to achieve 5 pm resolution for the bond length of N_2_ and O_2_. The retrieved N_2_ bond length is in agreement with the bond length of the neutral as well as the $N_{2}^{+}$ ion since they differ only by 2 pm. For O_2_, the retrieved bond length was 10 pm less than the bond length of O_2_. The difference was interpreted as due to the shrinking of the distance of the two oxygen atoms after an electron was removed by tunnel ionization, within the 5 fs for the electron to return to recollide with the $O_{2}^{+}$ ion. This interpretation is based on classical rescattering theory. It provides the first experimental evidence of bond length change at the sub-Ångström level within about 5 fs, the latter is the optical period of the 2 *μ*m laser, since ionization and recollision occur within one optical cycle. Very recently, Pullen *et al.*^24^ reported LIED experiments using 160 kHz, 3.1 *μ*m lasers to linear C_2_H_2_ molecules. They were able to extract both the C-C and C-H bond lengths based on the LIED method. In this experiment, the molecules are aligned and the retrieved bond lengths do not differ from the two alignments when molecules are parallel or perpendicular with respect to the laser polarization. In Pullen *et al.*, the ability to extract C-H bond length is unique for the LIED method since in LIED the electron energies used in these experiments are below 100 eV. Other ED experiments normally use keV electrons. At such high energies only heavy atoms are contributing to the DCS thus light atoms are generally not retrievable. In LIED, the use of lower electron energies and large scattering angles allows the retrieval of both the C-C and C-H bond lengths from the diffraction images in the region where the DCS from carbon and from hydrogen are comparable.

In the present paper, we demonstrate structure retrieval using LIED for a larger polyatomic molecule, benzene (C_6_H_6_). Unlike the unique home-built laser used in Pullen *et al.* which used the new OPCPA laser technology, we used the 1.65 *μ*m, 1 kHz laser that was obtained with the more common OPA laser technology. Such lasers are accessible today in most ultrafast laser laboratories. As a first test of LIED experiment in our laboratory, we chose benzene molecules which, like C_2_H_2_, also consist of only two kinds of atoms, C and H, but it has many more pairs of bond lengths that contribute to the diffraction images. If the benzene ring structure is substantially altered by the strong field ionization, then the diffraction images would not be accurately fitted by two bond lengths, C-C and C-H, only. Thus, the system allows us to test conditions that are favorable for LIED experiments.

## II. EXPERIMENTAL SETUP

**FIG. 2.**
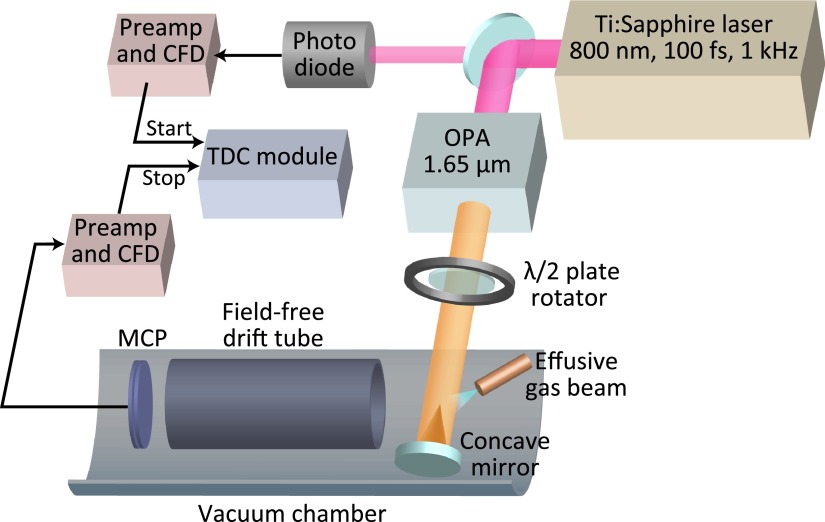
Schematic experimental setup for LIED.

A schematic configuration of the experiment is depicted in Fig. 2. We use a near-infrared Ti:Sapphire femtosecond laser system (0.8 *μ*m, 1.5 mJ/pulse, 100 fs, 1 kHz) to pump an optical parametric amplifier (OPA) to generate tunable short linearly polarized infrared pulses with a duration of about 100 fs. The IR laser beam of the idler output of the OPA at a wavelength of 1.65 *μ*m with a laser intensity around 120 *μ*J/pulse is focused by an *f* = 75 mm reflective spherical mirror in an ultra-high-vacuum chamber with a base pressure of 2 × 10^−10 ^ Torr. Benzene vapor was effusively introduced into the chamber by a needle valve. The working pressure of the chamber is about 10^−6^–10^−8 ^ Torr, but the pressure in the laser focus is estimated to be 10−20 times that of the background working pressure. We measure the angle-resolved high-energy photoelectron spectra of randomly oriented benzene molecules with a 264 mm long linear time-of-flight spectrometer. The photoelectrons are detected by a microchannel plate (MCP) followed by a single metal anode. The acceptance angle of the spectrometer is about 0.0014 × 4*π*sr. The time difference between the laser pulse (recorded by a photodiode) and the arrival time of the electron was recorded by a time to digital converter with a time resolution of 0.5 ns, which limits the energy resolution of the photoelectron spectra. The energy resolution at 100 eV, Δ*E*/*E*, is around 1/45, and as the energy increases, the resolution decreases. The polarization direction of the laser pulse is rotated using a *λ*/2 plate. During the measurement, the *λ*/2 plate is rotated with a constant speed (one rotation per one minute) for hundreds times. This method can effectively avoid the laser power variation effect on the angular distribution of the low-count high-energy photoelectrons.

## III. RESULTS AND DISCUSSION

A typical example of the two-dimensional (2D) photoelectron momentum distributions is given in Fig. 1(b), where the laser polarization direction is along the horizontal axis and the vertical axis is along any direction perpendicular to it (due to cylindrical symmetry in the measurement). We have extracted the DCSs according to the QRS theory. More details are given in the supplementary material.

The data were taken for a laser intensity of 1.1 × 10^14 ^W/cm^2^ in order to obtain better statistics. The count rate was up to ∼120 cps for backward rescattered electrons at 180∘, and the total accumulation time was 6 h. We limit the analysis to the inner part of the spectrum where the returning electron momentum (*p_r_*) is below 2.1 a.u., or energy around 50–65 eV. We exclude images from higher momenta since they arise from the higher intensity region where multiple ionization has likely occurred. The extracted molecular DCSs (*σ*) are plotted in Fig. 1(c), together with the calculated atomic DCSs (*σ_a_*), as a function of the rescattering angle (*θ_r_*) for *p_r_* = 2.0 a.u. Here, the atomic DCS is the incoherent sum of DCSs of all the atoms in the molecule. The extracted molecular DCS oscillates about the smooth atomic DCS curve. According to IAM, the oscillatory difference contains the molecular structure information. We define the molecular contrast factor (MCF)

$$MCF=\frac{\sigma -\sigma_{A}}{\sigma_{A}}$$ (1)

as a function of momentum transfer $s=2p_{r}\;\text{sin}(\theta_{r}/2)$. Since the returning electron flux depends on the orientation-dependent tunnel ionization rate, both the atomic and molecular DCSs above have to be convoluted over the randomly distributed molecules weighted by the angle-dependent ionization rate. These ionization rates can be calculated using the molecular tunneling theory (MO-ADK)^33^ or with the strong-field approximation (SFA). The two methods give nearly identical results.

**FIG. 3.**
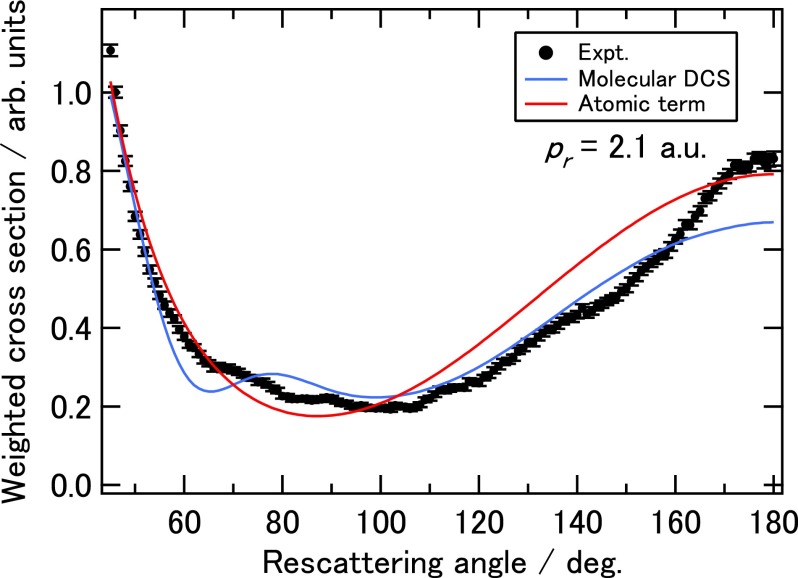
Weighted elastic scattering cross section extracted from the experimental 2D momentum spectra for recolliding momentum of 2.1 a.u. (or electron recollision energy of 60 eV). Theoretical molecular DCS calculated using IAM with equilibrium geometry (solid blue line) and the atomic DCS (solid red line) are also shown.

A typical experimentally extracted DCS vs rescattering angle (*θ_r_*) is shown in Fig. 3 for electron momentum (*p_r_*) of 2.1 a.u. The atomic term is also shown. The error bars are calculated as the square roots of the electron counts for statistical errors. We remark that at these low collision energies, the DCS from hydrogen is comparable to that from carbon.^24^ Clearly, the experimental DCS oscillates around the atomic term. A similar oscillation can also be seen in the theoretical DCS calculated using the IAM for benzene with its equilibrium geometry, shown in blue line in Fig. 3. There is a fair overall agreement between theory and experiment except for angles larger than 160∘. Such discrepancy was seen in Pullen *et al.*^24^ as well since in both experiments long pulses are used. We do not use data for angles larger than 160∘. The reason is explained in the supplementary material, and it would not affect our structure retrieval.

**FIG. 4.**
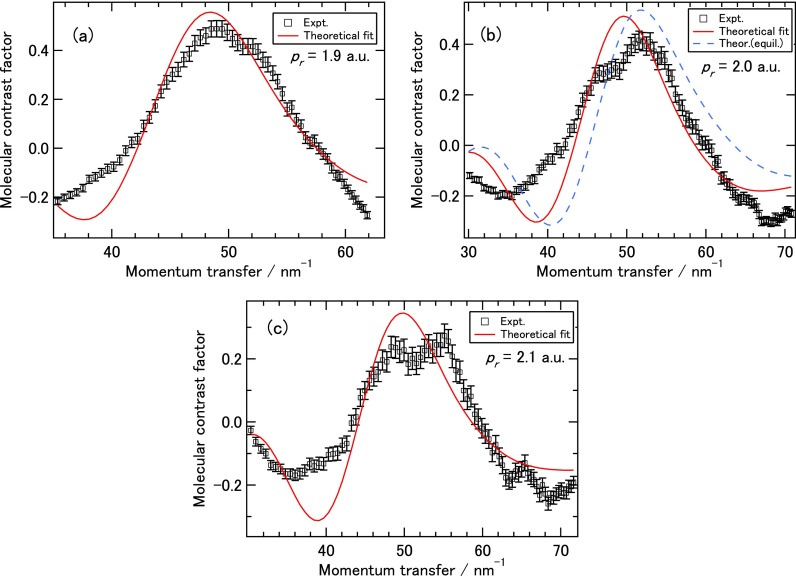
Experimental molecular contrast factor (MCF) (symbols) and optimally fitted theoretical MCF with bond lengths as fitting parameters (solid red curve) for recolliding momentum *p_r_* of 1.9 a.u. (a), 2.0 a.u. (b), and 2.1 a.u. (c). The theoretical MCF corresponding to the equilibrium geometry is also shown for *p_r_* = 2.0 a.u. in (b) (dashed blue curve).

To retrieve bond lengths from the experimental DCS, we employed the procedure proposed by Xu *et al.*^22^ which was used by Blaga *et al.*^23^ and Pullen *et al.*^24^ Specifically, we used C-C and C-H bond lengths as fitting parameters such that the theoretical MCFs best agree with the experimental ones. The use of MCF for fitting is preferable since it is proportional to the interference term. In actual retrieval we fit the MCF in the angular range of 40∘–140∘ only. Results from such fitting are shown in Figs. 4(a)–4(c) for electron momentum (*p_r_*) of 1.9, 2.0, and 2.1 a.u., respectively. The corresponding retrieved C-C and C-H bond lengths are 152 pm and 113 pm for *p_r_* = 1.9 a.u., 147 pm and 114 pm for *p_r_* = 2.0 a.u., and 143 pm and 126 pm for *p_r_* = 2.1 a.u., respectively. These values are within about 10% of the equilibrium ones of 139 pm and 109 pm, for the C-C and C-H bond length, respectively. With these retrieved parameters, the quality of the overall MCF fitting is relatively good in the range of momentum transfer from about 30 to 70 nm^−1^. Note that this range is quite typical of current LIED experiments with polyatomic molecules (see, for example, Ref. 24) and for X-ray experiments from LCLS.^13^ In fact, the fitting nicely reproduces the strong peaks near momentum transfer *s* ≈ 50 nm^−1^, but the fitted peak position is slightly lower than the experimental one for each of the three energies considered here. An example of chi-square fitting result is given in more detail in the supplementary material. To further judge the fitting procedure, we also show in Fig. 4(b) the theoretical MCF based on the IAM assuming that benzene remains at its equilibrium geometry (dashed blue curve). The fitting gets slightly better overall agreement with the experimental MCF, thus it may imply that there are some degrees of deformation of the molecule after tunnel ionization. This is also consistent with the observation that the experimental MCF flattens out as the returning electron momentum increases. However, to draw firm conclusion on this speculation it would need more experimental data and a direct structure retrieval method since convergence in many-parameter fitting method is often challenging. To this end, in the future it is desirable to have diffraction images from 1D aligned molecules from which 2D molecular structure (including bond angles) can be retrieved. For LIED, this was addressed recently in Yu *et al*.^34^ Experimentally, it is also desirable to measure photoelectron angular distributions in coincidence with molecular ions.^24^

## IV. CONCLUSION

In summary, we have measured photoelectron angular distributions of benzene molecules ionized by 1.65 *μ*m, 100 fs infrared laser pulses. We demonstrated that accurate C-C and C-H bond lengths can be retrieved using the laser-induced electron diffraction method (LIED) by analyzing diffraction images generated by returning electrons with momenta close to about 2.0 a.u. The retrieved data are consistent with the model that the benzene ring remains intact. Our results demonstrate that most typical ultrafast intense laser laboratories today have the capability of carrying out LIED experiments to extract positions of atomic constituents in a molecule with sub-Ångström spatial resolution. The LIED method relies on nonlinear ionization of molecules by intense laser pulses. For each molecular system, it has a range of laser parameters where LIED will work best. For light molecules, LIED has demonstrated its ability to achieve sub-Ångström spatial resolution. Since femtosecond laser pulses are used in the probing, temporal resolution of tens of femtoseconds is already given.

Looking ahead, the challenge for LIED as a potential powerful table-top laboratory tools for ultrafast dynamic imaging is whether good quality diffraction images can be generated at a typical laser laboratory like ours. The result reported here is definitely a positive statement. Still the technology has room to improve. Future LIED experiments should include oriented/aligned targets^24,35–38^ and coincidence of photoelectrons with molecular ions.^24^ In terms of lasers, shorter pulses and longer wavelength are desirable such that high energy electrons are generated without excessive ionization. Higher flux of returning electrons is also desirable and it can be met in the future with high-repetition (hundreds kHz and up) lasers, or few-color synthesized laser that optimizes the fraction of returning electrons without excessive ionization (see Jin *et al.*^39^ and references therein). For LIED to become a competitive ultrafast dynamic imaging tool for molecules, more typical ultrafast laser laboratories should be engaged in such investigations.

## V. SUPPLEMENTARY MATERIAL

See supplementary material for the detail of the QRS theory, DCS extraction, and chi-square fitting.
